# Characterization of *SALL2* Gene Isoforms and Targets Across Cell Types Reveals Highly Conserved Networks

**DOI:** 10.3389/fgene.2021.613808

**Published:** 2021-02-22

**Authors:** Carlos Farkas, Aracelly Quiroz, Claudia Alvarez, Viviana Hermosilla, Carlos F. Aylwin, Alejandro Lomniczi, Ariel F. Castro, Matias I. Hepp, Roxana Pincheira

**Affiliations:** ^1^Laboratorio de Transducción de Señales y Cáncer, Departamento de Bioquímica y Biología Molecular, Facultad de Ciencias Biológicas, Universidad de Concepción, Concepción, Chile; ^2^Division of Neuroscience, Oregon National Primate Research Center, Oregon Health and Science University, Portland, OR, United States; ^3^Laboratorio de Investigación en Ciencias Biomédicas, Departamento de Ciencias Básicas y Morfología, Facultad de Medicina, Universidad Católica de la Santísima Concepción, Concepción, Chile

**Keywords:** SALL2, isoforms, transcription factor network, PODXL, ENCODE ChIP-seq, Glioblastoma, stem cell transcription factors

## Abstract

The SALL2 transcription factor, an evolutionarily conserved gene through vertebrates, is involved in normal development and neuronal differentiation. In disease, SALL2 is associated with eye, kidney, and brain disorders, but mainly is related to cancer. Some studies support a tumor suppressor role and others an oncogenic role for SALL2, which seems to depend on the cancer type. An additional consideration is tissue-dependent expression of different SALL2 isoforms. Human and mouse *SALL2* gene loci contain two promoters, each controlling the expression of a different protein isoform (E1 and E1A). Also, several improvements on the human genome assembly and gene annotation through next-generation sequencing technologies reveal correction and annotation of additional isoforms, obscuring dissection of SALL2 isoform-specific transcriptional targets and functions. We here integrated current data of normal/tumor gene expression databases along with ChIP-seq binding profiles to analyze SALL2 isoforms expression distribution and infer isoform-specific SALL2 targets. We found that the canonical SALL2 E1 isoform is one of the lowest expressed, while the E1A isoform is highly predominant across cell types. To dissect SALL2 isoform-specific targets, we analyzed publicly available ChIP-seq data from Glioblastoma tumor-propagating cells and in-house ChIP-seq datasets performed in SALL2 wild-type and E1A isoform knockout HEK293 cells. Another available ChIP-seq data in HEK293 cells (ENCODE Consortium Phase III) overexpressing a non-canonical SALL2 isoform (short_E1A) was also analyzed. Regardless of cell type, our analysis indicates that the SALL2 long E1 and E1A isoforms, but not short_E1A, are mostly contributing to transcriptional control, and reveals a highly conserved network of brain-specific transcription factors (i.e., SALL3, POU3F2, and NPAS3). Our data integration identified a conserved molecular network in which SALL2 regulates genes associated with neural function, cell differentiation, development, and cell adhesion between others. Also, we identified *PODXL* as a gene that is likely regulated by SALL2 across tissues. Our study encourages the validation of publicly available ChIP-seq datasets to assess a specific gene/isoform’s transcriptional targets. The knowledge of SALL2 isoforms expression and function in different tissue contexts is relevant to understanding its role in disease.

## Introduction

In humans, the *SALL2* gene is located on chromosome 14 region 14q11.1- 12 (Kohlhase et al., 1996). This region was initially associated with loss of heterozygosity in ovarian cancer (Bandera et al., 1997), where later it was characterized as a tumor suppressor (Li et al., 2004). Accordingly, SALL2 upregulated p21 and BAX tumor suppressors in ovarian cancer cells and repressed c-MYC oncogene under genotoxic stress (Li et al., 2004; Gu et al., 2011; Sung et al., 2012). Also supporting its role as a tumor suppressor, SALL2 upregulated Phorbol 12-Myristate 13 Acetate-Induced Protein-1 (*PMAIP-1* also known as Noxa) under genotoxic stress in Jurkat T- acute lymphoblastic leukemia cells and repressed cyclins D1 and E1 during G1/S transition in mouse embryonic fibroblasts (Escobar et al., 2015; Hermosilla et al., 2018). Previous studies described two main SALL2 isoforms, the canonical isoform (SALL2 E1) and the alternative isoform (SALL2 E1A). These isoforms share a long common exon but differ in short exons and are encoded from alternative use of promoters located 11 kilobases away one from another. Thus, the SALL2 E1 (1007 residues) and SALL2 E1A (1005 residues) isoforms only differ in 25 and 23 residues at the N-terminal, respectively (Ma et al., 2001). Currently, the human *SALL2* gene has seven protein-coding transcripts annotated in Ensembl composed by a combination of at least five exons^1^ (December 2019). This updated information poses the questions of which isoform(s) is (are) predominant in every cell type or tissue studied, and which is (are) responsible for gene expression.

Recent advances in high-throughput technologies have facilitated studies of genome-wide alternative splicing. Roughly half of the human genome’s expressed genes displayed tissue-dependent transcript isoforms due to alternative transcription sites usage, rather than alternative splicing (Reyes and Huber, 2018). This transcript diversity is essential for regulating various biological processes (Pozner et al., 2007; Gabut et al., 2011; Schaub and Glasmacher, 2017), and aberrant expression of transcripts is associated with diseases such as cancer (Mayr and Bartel, 2009; Xiong et al., 2015; Sendoel et al., 2017). The availability of high-quality tumor RNA sequencing provided by international consortia such as the Cancer Genome Atlas (TCGA) (Cancer Genome Atlas Research Network et al., 2013), International Cancer Genome Consortium (ICGC) (Zhang et al., 2011), Genotype-Tissue Expression (GTEX) (GTEx Consortium, 2013) and Broad Institute Cancer Cell Line Encyclopedia (CCLE) (Barretina et al., 2012), enables robust characterization and discovery of cancer/tissue-specific isoforms, which can be explored in various ways and can be intersected with next generation sequencing data (Niknafs et al., 2018). Additionally, chromatin immunoprecipitation sequencing (ChIP-seq) can be used for inferring regulatory pathways and the control of gene expression under disease or in a given cell-type/tissue.

Here, we integrated the isoform-specific expression of SALL2 across many normal/tumor tissues with RNA seq datasets from three SALL2 isoforms (E1, E1A, short_E1A). Also, we integrated publicly available datasets from glioblastoma MGG8 tumor propagating cells (MGG8TPC) SALL2 ChIP-seq and HEK293 expressing SALL2 short_E1A ChIP-seq (ENCODE Consortium Phase III), and in-house ChIP-seq datasets from SALL2 wild-type and E1A isoform knockout HEK293 cells. We found a network of a core of transcription factors (TF) accompanying SALL2 in the control of gene expression and concluded that the SALL2 long E1 and E1A isoforms are most likely contributing to transcriptional control. We propose minimal contribution of the short_E1A isoform in these networks, due to its low connected gene network, low binding occupancy in the genome, and cytoplasmic localization. Moreover, we observed low expression of the rest of the SALL2 reported isoforms across tissues in Ensembl. Importantly, SALL2 regulates the same core of genes in glioblastoma TPC and HEK293 cells, sustaining a conserved network of SALL2 target genes. Our work provides insights into SALL2 isoform-specific transcriptional targets and evidence of a conserved TF network relevant to cancer studies.

## Materials and Methods

### Isoform Quantification Across Human Normal/Tumor Datasets

*SALL2* gene counts across normal/tumor including associated statistics (*p*-value calculation) in tumor/normal gene quantifications were downloaded from Michigan Portal for the Analysis of NGS data portal^2^ (Niknafs et al., 2018), which combines Transcript per million values (TPM) from next-generation sequencing datasets provided from TCGA, ICGC, GTEX, and CCLE consortia (Zhang et al., 2011; Barretina et al., 2012; Cancer Genome Atlas Research Network et al., 2013; GTEx Consortium, 2013). *SALL2* isoform-specific counts were obtained from ISOexpresso server, containing spliced RNA-seq data obtained from TCGA (Yang et al., 2016). These data-associated plots were performed in R packages such as pheatmap^3^, tidyverse^4^, including in-house R scripts. Most of the computational analysis required to reproduce the manuscript analysis are available in this repository: https://github.com/cfarkas/SALL2_conserved_network_analysis.

### ChIP-Seq

Chromatin immunoprecipitation was carried out as previously described (Henriquez et al., 2011), with the following modifications: HEK293 cells (*SALL2* wild-type, *SALL2* E1A-knockout, and *SALL2* total knockout) were grown in 10 cm dishes at 1 × 10^6^ cells per plate. Cell nuclei were sonicated to shear DNA in 300 μl of sonication buffer, using a Bioruptor Plus (Diagenode) (18 times, 15 s on /20 s off each time, 9 W potency), obtaining fragments between 300 and 600 bp after 12 cycles of PCR amplification. Immunoprecipitations (IP) were carried out overnight at 4°C using 40 μg of chromatin and 5 μg of Rabbit Polyclonal anti-SALL2 (Bethyl Cat# A303-208A, RRID:AB_10953171). The IP in *SALL2* total knockout cells was used as a negative control of antibody binding. Each library was prepared using the KAPA HypperPrep kit (Roche) and seven adapters from the KAPA SI Adapters kit, according to the manufacturer’s protocols. Briefly, the first step in library preparation was to convert any overhangs in the ChIP DNA into phosphorylated blunt ends. The 3′ ends were then adenylated, and adaptors ligated onto the ends of the fragments. The library average size (348 bp) and the DNA concentration were obtained using an Agilent 2100 Bioanalyzer. Each library was sequenced using an Illumina HiSeq 4000 machine at Genomics & Cell Characterization Core Facility of the University of Oregon, Portland, OR, United States.

### ChIP-Seq Analysis

Illumina ChIP-seq reads of *SALL2* wild-type, *SALL2* E1A-knockout cells, and SALL2 total knockout HEK293 cells (GEO datasets GSE145940) were imported to the Galaxy platform (Giardine et al., 2005) and aligned to the human genome (hg19 assembly) by using bowtie2 aligner with default settings (Langmead and Salzberg, 2012). Correspondent BAM files were sorted (Galaxy Version 1.7), and alignments filtered with a sliding quality window of Q20 by using SAMtools (Galaxy Version 1.7) (Li et al., 2009). We used MACS2 program to call peaks in these BAM files by using a significant genome size of 2,700,000,000 base pairs, read extension size of 151, and peak detection based on *p*-value cutoff of 0.005. Additionally, bigwig files from aligned bam files were obtained using the deeptools bam Coverage tool (Galaxy Version 3.1.2) (Ramirez et al., 2016). Each bigwig file was normalized to 1x method using the referred significant genome size and a bin length of 50 bp. A matrix was then computed for each dataset with a deeptools computeMatrix tool, according to the sort of the ChIP-seq peaks by using BED files of 2-kb window, centered to the center of the peak in each case. Finally, each dataset’s heatmaps were plotted with the deeptools plot Heatmap tool maintaining the bed coordinates’ ordering. The colormap used in the heatmaps was jet arrays, and the missing data color in the plots was dark blue. ChIP-seq peaks in BED format were annotated using the Bioconductor package ChIPseeker in R software^5^ (Yu et al., 2015). Motif Analysis across MGG8TPC, short_E1A (ENCODE) WT, and E1 ChIP-seq datasets were obtained with the RSAT program^6^ (Thomas-Chollier et al., 2008). The top three over-represented oligos per dataset were selected based on *p*-value and recurrence across nucleotide sequences from peaks. To obtain transcription factor network enrichments by orthogonal omics integration, resulting in annotated gene lists for every ChIP-seq dataset were submitted to the online server ChEA3 (Keenan et al., 2019). GO enrichment analysis from the gene-annotated outputs from ChIPSeeker in every ChIP-seq analyzed was performed by submitting these list to the gProfiler server (Reimand et al., 2007) and selecting GO terms using a false discovery rate (FDR) cutoff of 0.05. ChIP-seq tracks in BigWig format including associated BED files containing peaks were visualized with the IGV software (Robinson et al., 2011).

#### Publicly Available ChIP-Seq Data Sets Analyzed

We selected from GEO datasets available ChIP-seq experiments that involve SALL2 as the principal target of DNA binding. (1) The GSE54047 study performed in MGG8TPC that used the SALL2 antibody (Bethyl laboratory) for endogenous SALL2 binding, (2) the GSE105193 study from HEK293 stably expressing N-terminal eGFP-SALL2 fusion protein (short-E1A) and the eGFP antibody (Abcam) to detect SALL2 binding and (3) ChIP-seq of H3K27ac, H3K4me3, and H3K27me3 histone marks in HEK293 submitted by ENCODE and independent laboratories. A complete description of these datasets, including the associated computational analysis, is available here: https://github.com/cfarkas/SALL2_conserved_network_analysis. Methods to integrate these analyzes are summarized in Supplementary Figure 1.

### Quantitative Real-Time Reverse Transcription-PCR Assay

Cells were collected, and total RNA was isolated using TRIzol (ThermoFisher Scientific, Inc.) and conventional Phenol/chloroform/isoamyl alcohol extraction method (in ratio 25:24:1, respectively) (Toni et al., 2018). The RNA samples were treated with Turbo DNA-free Kit (Invitrogen) to eliminate any residual DNA from the preparation. Total RNA (2 μg) was reverse transcribed using the M-MLV reverse transcriptase (PROMEGA) and 0.25 μg of Anchored Oligo(dT)20 Primer (Invitrogen; 12,577–011). We performed qPCR reactions using KAPA SYBR FAST qPCR Master Mix (2X) Kit (Kapa Biosciences) with primer concentrations of 0.4 μM. The primers used in the reactions are listed in Supplementary Table 1. Cycling conditions were as follows: initial denaturation at 95°C for 3 min, then 40 cycles with 95°C for 5 s (denaturation) and 60°C for 20 s (annealing/extension). The melting curve indicates no amplification of unspecific products. The expression of each gene was relative to the *PPIB* gene (cyclophilin).

### Nuclear/Cytoplasmic Fractionation and Western Blot Analysis

HEK293 KO cells infected with short_E1A isoform were grown to ∼70% confluence in 10-cm dishes and collected in ice-cold Phosphate Buffered Saline solution (PBS). Then, cells were centrifuged at 1,000 rpm for 5 min, and the supernatant was removed. Cell pellets were processed with the Nuclear/Cytosol Fractionation Kit according to the manufacturer instructions (BioVision, Inc., Cat # K266). Nuclear and cytoplasmic fractions, and whole cell lysates (50–80 μg of total protein) were fractionated by SDS-PAGE and transferred for 1 h at 200 mA to PVDF membranes (Immobilon; Millipore). PVDF membranes were blocked for 1 h at room temperature in 5% non-fat milk in TBS-T (TBS with 0.1% Tween) and incubated with primary antibody at appropriate dilution in TBS-T 4°C overnight. After washing, the membranes were incubated with horseradish peroxidase-conjugated secondary antibodies diluted in TBS-T buffer for 1 h at room temperature. Immunolabeled proteins were visualized by ECL (General Electric Healthcare, Amersham, United Kingdom). Antibodies used for Western blotting were as follows: monoclonal anti-FLAG, Clone M2 (1:1500, Sigma-Aldrich Cat# F3165, RRID:AB_259529), monoclonal anti-MEK-1 (1:500, Santa Cruz Biotechnology Cat# sc-6250, RRID:AB_627922), polyclonal anti-Lamin B1 (1:500, Abcam Cat# T1507, RRID:AB_10705085), monoclonal anti-β-actin (1:10000, Santa Cruz Biotechnology Cat# sc-47778 HRP, RRID:AB_2714189), and polyclonal anti-SALL2 (1:1000, ATLAS antibodies, HPA004162; Millipore).

### Cloning of Short_E1A SALL2 Isoform

Short-E1A *SALL2* isoform (GenBank: BC024245.2) was synthesized (GenScript) with SalI and AgeI sites in the 5′ and 3′ ends. The 651 bp of cDNA was subcloned using AgeI and SalI restriction enzymes and the lentiviral vector pCW57-MCS1-2A-MCS2 (Addgene, plasmid 71782).

### Cell Culture, Transient Transfections, and Viral Infection

HEK293 (ATCC Cat# CRL-1573, RRID:CVCL_0045), MCF7 (ATCC Cat# HTB-22, RRID:CVCL_0031), HCT116 (NCI-DTP Cat# HCT-116, RRID:CVCL_0291), LS174T-HM7 (RRID:CVCL_6670), and CCD-841-CoN (ATCC Cat# CRL-1790, RRID: CVCL_2871) cell lines were grown in Dulbecco’s modified Eagle’s medium (DMEM) (Hyclone, Logan, UT, United States) supplemented with 10% (v/v) fetal bovine serum (FBS; Hyclone), 1% glutamine (Invitrogen Santa Fe, Mexico DF, Mexico) and 1% penicillin/streptomycin (Invitrogen), in 10 cm plates. H1299 cell line (NCI-DTP Cat# NCI-H1299, RRID:CVCL_0060) were grown in RPMI 1640 media (Hyclone, Logan, UT, United States) with the same supplements used in DMEM media. A total of 1.5 × 10^6^ cells were transfected with 30 μg of plasmids using Lipofectamine 2000 reagent (Invitrogen, Life Technologies). For viral infection of short_E1A *SALL2* isoform, lentivirus was packaged by co-transfection of constructs pCMV-dR8.2 dvpr (Addgene, plasmid_8455), pCMV-VSVG (Addgene, plasmid_8454) and pCW57-MCS1-2A-MCS2 (Addgene, plasmid_71782) containing or not the referred cDNA isoform. After transfection, the medium was changed every 24 h with 9 μg/ml of polybrene, and the 24, 48, and 72-h supernatants were filtered through a 0.45 μm filter and added to *SALL2* knockout HEK293 cells. These cells were selected with 6 μg/ml of puromycin and recovered with fresh DMEM medium.

### CRISPR-Cas9 Knockout Generation

HEK293 and CCD-841-CoN *SALL2* total knockout clones were obtained by CRISPR-Cas9, as described in Escobar et al. (2015) and Hermosilla et al. (2018). The HEK293 *SALL2* E1A-knockout cell model was obtained by electroporation at 1100 volts per 20 milliseconds (NEON Transfection System, Thermo Fisher Scientific), with a vector encoding Cas9, a specific *SALL2* RNA guide (pX330, Addgene), and GFP or RFP-containing vector used as a transfection marker. Control cells were electroporated using the GFP or RFP-containing vector alone. Supplementary Table 1 indicates the specific human *SALL2* guide RNAs and primers used for PCR reactions and sequencing. We collected 1000 cells by GFP sorting from which ten clones were obtained. From three clones, genomic DNA was purified and sequenced, giving one clone with the expected deletion (clone 17). Validation of SALL2 E1A included Sanger sequencing (performed at Pontificia Universidad Católica Sequencing Facility, Santiago, Chile) and Western Blot analysis (Supplementary Figures 2, 3).

### Immunocytochemistry

Cells were seeded on polylysine-coated coverslips. After fixation with 4% paraformaldehyde, cells were permeabilized with 0.1% Triton X100 in PBS for 10 min and rinsed twice with PBS. Coverslips were incubated at 4^o^ C overnight with primary antibodies (anti SALL2 or anti FLAG) diluted in 3% BSA, followed by a 2-h incubation with Hoechst 33342 and Alexa fluor-conjugated secondary antibodies. Images were obtained with LMS 780 spectral confocal system (Zeiss, Jena, Germany) in Centro de Microscopía Avanzada CMA BIO BIO (Proyecto ANID PIA ECM-12), Facultad de Ciencias Biológicas, Universidad de Concepción, Concepción, Chile. Each experiment was repeated at least three times.

## Results

### *SALL2* Locus Expresses at Least Seven Tissue-Specific Isoforms, Supported by Orthogonal Omics Integration

**FIGURE 1:**
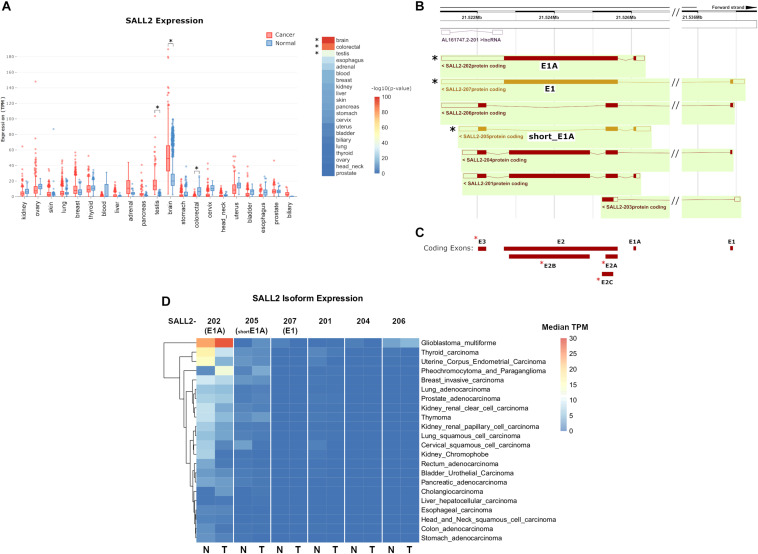
**(A)** (Left) boxplot of *SALL2* gene expression levels in a panel of normal and cancer tissue counterparts obtained from the MiPanda server (http://www.mipanda.org/). RNA-sequencing reads were quantified to the human transcriptome (GENCODEv25) using Kallisto (v0.43.0), and gene-level expression was obtained by summing the TPM values for all *SALL2* transcripts in each plotted dataset. Plotted datasets combine TCGA, GTEX, and CCLE databases. (Right) -log10(*P*-Value) of the *T*-test between Normal and Cancer TPM values. Brain and colorectal values are denoted due to high significance (asterisks). **(B)** Screenshot of the Ensembl (v98) browser depicting *SALL2* locus and its annotated isoforms obtained from automatic annotation and Havana manual curation (Orange = merged annotation, Red = Havana annotation). Seven isoforms are identified, including the known *SALL2* canonical isoform (E1), E1A, and a short isoform from E1A promoter (short_E1A) indicated with asterisks. **(C)** Exon models from *SALL2* locus derived from the above isoforms. The three known exons are denoted (E1, E1A, and E2) plus novel proposed exons (red asterisks). **(D)** Plot of *SALL2* mRNA isoform expression across normal and cancer tissues. E1, E1A, and short_E1A isoforms are depicted in parentheses. Average median TPM values per isoform were obtained from the ISOexpresso server (http://wiki.tgilab.org/ISOexpresso/) and plotted in R. Scale values in TPM are colored as depicted in the figure.

Previous studies based on a limited number of samples indicate that the *SALL2* gene is highly expressed in the normal brain, testis, thymus (Ma et al., 2001), and differentially expressed in some cancers (Hermosilla et al., 2017). Interestingly, *SALL2* is absent or low expressed in ovarian cancer, whereas it is highly expressed in Glioblastoma context (Suvá et al., 2014). To further investigate *SALL2* expression in normal and cancer contexts based on more extensive studies, we used MiPanda server. This open-access online resource provides access to the results of a large-scale computational analysis of thousands of high-throughput RNA sequencing (RNA-seq) samples (Niknafs et al., 2018). Distribution of *SALL2* expression in human normal tissues confirmed the highest expression levels in the normal brain and pituitary gland (Supplementary Figure 4 and Supplementary Table 2, raw values). Analyses of expression patterns from cancer samples versus normal samples indicated that *SALL2* has differential expression in 16 out of 21 cancer types, most significantly in the brain, colorectal, and testis tissues (Supplementary Table 2, *p*-values). *SALL2* expression is upregulated in brain and testis tumors (*p*-value 1.66e-99 and 1.29e-40, respectively) but significantly downregulated in colorectal cancer tumors (*p*-value 1.72e-90) (Figure 1A, asterisk).

To dissect tissue-specific *SALL2* isoforms expression, we analyzed the annotated isoforms from Ensembl (December 2019). Seven isoforms and one long non-coding RNA are associated with the *SALL2* locus (Figure 1B), with at least seven exons usage (Figure 1C). This number exceeds the three known exons of the *SALL2* gene (including three novel alternative exons for exon E2 and a novel exon E3) (Figure 1C, red asterisks), and explains the transcript diversity across tissues observed in Ensembl. Among the seven isoforms, we identified the E1 and E1A isoforms, and a short version of E1A (Figure 1B, black asterisks). We observed low expression of the rest of the SALL2 isoforms across tissues (Supplementary Figure 5). Additionally, the E1 isoform, and the short version of E1A (named hereafter short_E1A) were manually curated by the HAVANA project, supporting their existence^7^. As expected from previous reports (Ma et al., 2001), transcript per million (TPM) counts of each isoform across tissues revealed that the *SALL2* E1A isoform is highly predominant. Surprisingly, the canonical E1 isoform has nearly zero TPM counts, and the short_E1A surpasses any other isoform expression levels, except the long E1A isoform expression (Supplementary Figure 5). Spliced RNA-seq data from the ISOexpresso database (Yang et al., 2016) showed that the E1 isoform expression across samples is strictly restricted to the normal brain (Figure 1D, see SALL2-207). The E1A isoform expression differs between many normal and cancer tissues, except in hepatocellular carcinoma (Figure 1D, see SALL2-202). According to the overall *SALL2* expression, the E1A isoform is highly predominant in brain cancer and absent in colon cancer, supporting its association with these cancers. The short_E1A isoform expresses at a similar level as the E1A isoform in some tissues, but at a lower level in others such as thyroid, breast, and uterine tissues (Figure 1D, see SALL2-205). Similar to the E1A isoform, the short_E1A isoform is also differentially expressed in brain cancer.

Interestingly, in glioblastoma multiforme (GBM), E1A and short_E1A are upregulated, but E1 is downregulated (Figure 1D). Altogether, our analysis indicates that the transcript diversity and exon usage in the *SALL2* locus exceed the previous studies and that the SALL2 E1A isoform is the most expressed. Interestingly, the novel short_E1A isoform greatly surpasses the canonical SALL2 E1 isoform expression, suggesting a biological role.

### SALL2 Short_E1A Isoform Is Functionally Unrelated to the Other SALL2 Isoforms and Localizes in the Cytoplasm

**FIGURE 2:**
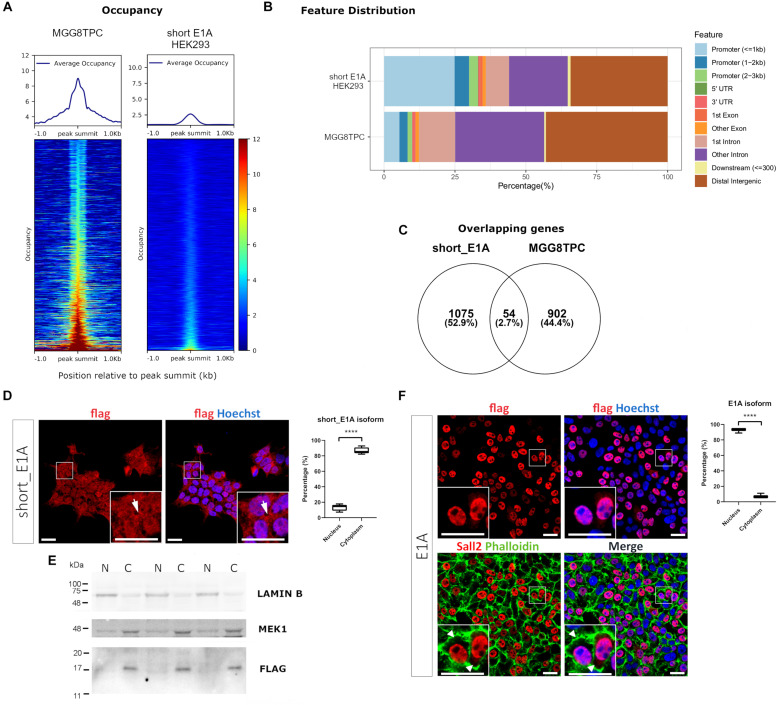
**(A)** Plot of the average occupancy and heatmap of the occupancy profiling from two publicly available SALL2 ChIP-seq datasets, sorted by occupancy values from the smallest (top) to highest (bottom). Each row represents a 1-kb window focused on the peak centers in the human genome (hg19 build). (Left) Plot of 1328 peak regions from a reported in a subset of stem-like tumor-propagating cells in Glioblastoma Multiforme (MGG8TPC) SALL2 ChIP-seq using a human anti-SALL2 antibody (Bethyl Laboratories) (GEO datasets GSE54047). (Right) Plot of 1484 peak regions from a reported eGFP-short_E1A SALL2 ChIP-seq done on HEK293 cells, using an anti-GFP antibody, provided by ENCODE consortium (GEO datasets GSE105193). BigWig files used in both plots were generated using a 1x-normalized method against hg19 build, and values were plotted in the heatmaps using the same color scale for comparison purposes. **(B)** Feature distribution of the binding sites of these two ChIP-seq (in percentage) upstream and downstream from the TSS of genes. Promoters, UTR, and exon regions are denoted with distinctive colors. **(C)** Venn diagram of the overlapping peaks between the two ChIP-seq datasets. **(D)** (Left), immunocytochemistry of HEK 293 cells infected with Flag-SALL2 short_E1A, detected with anti-Flag and Alexa fluor-conjugated antibodies. Short_E1A (Flag) shown in red, and nuclei (Hoechst-stained) is in blue. (Right) Quantification of Flag signal between Nucleus and Cytoplasm per cell, expressed as percentages per each isoform. The *T*-test (****p < 0.0001) is denoted with asterisks. **(E)** Nucleus/Cytoplasm fractioning of Flag-SALL2 short_E1A infected HEK293 cells. LAMIN B and MEK1 proteins are markers of the nucleus (N) and cytoplasm (C). Flag detects Short_E1A. **(F)** (Left) Flag-SALL2 E1A long isoform is detected as in **(D)**, phalloidin (F-actin-marker) is shown in green to denote cell boundaries since this isoform localizes mainly in the nucleus (see white arrowheads). (Right) Quantification of Flag signal between Nucleus and Cytoplasm per cell expressed in percentages as in **(D)**. **** *p* < 0.0001. Magnifications in immunocytochemistry are enclosed in squares at the bottom of each image for detailed visualization purposes. White bars denote 20 μM.

Because of the potential biological role of short_E1A, we initially investigate if this isoform is involved in transcriptional regulation by analyzing a ChIP-seq experiment performed in HEK293 cells provided by the ENCODE consortium (GEO datasets GSE105193^8^). An endogenous SALL2 ChIP-seq performed in stem-like tumor-propagating subpopulation from Glioblastoma Multiforme (hereafter MGG8TPC) was included as a binding positive control in this analysis (GEO datasets GSE54047). The normalized average occupancy around peaks from the SALL2 ChIP-seq in MGG8TPC revealed that SALL2 strongly binds above background (Figure 2A, left). Conversely, the short_E1A isoform density heatmap displays no enriched signal in more than half of regions (top half) suggesting low binding in the genome, like the background (Figure 2A, right). Peaks distribution in MGG8TPC showed preferential binding of SALL2 to introns/intergenic regions with a small subset of peaks in the promoter of genes (Figure 2B). This behavior was already reported for several transcription factors in the human genome (Farnham, 2009). Conversely, the short_E1A isoform peaks distributed more homogeneously across the human genome, indicating no differences between promoters and intergenic regions (Figure 2B).

We overlapped genes between the two datasets to identify potential transcriptional targets controlled by the short_E1A isoform, conserved in MGG8TPC. However, the overlaps revealed a low number of conserved target genes (around 3%, Figure 2C and Supplementary Table 3), suggesting that the short_E1A isoform is functionally unrelated to the other SALL2 isoforms in MGG8TPC. Because we compared transcriptional targets from two different tissues, the discreet overlapping may be due to tissue-specific transcription factor binding in the human genome (Spitz and Furlong, 2012). As previously reported, gene ontology analysis revealed various highly related neural functions of SALL2 isoforms in MGG8TPC, including neuron differentiation, neurogenesis, cell adhesion, among many others involved in brain development (Supplementary Figure 6A, left) (Suvá et al., 2014). However, the short_E1A isoform displayed a few unspecific gene ontology terms, revealing no apparent biological function in the nucleus (Supplementary Figure 6A, right). Also, as previously reported in MGG8TPC, SALL2 isoforms primarily binds A/T rich motifs corresponding to the SOX and POU family of transcription factors (Supplementary Figure 6B, upper panel) while the short_E1A isoform top hits are significantly recruited to C/A repetitions, not related to the SOX and POU transcription factors binding patterns (Supplementary Figure 6B, lower panel).

To understand if a transcription factor network is associated with SALL2 in the referred tissues, we performed transcription factor network enrichment by inputting the target gene list from the two ChIP-seq studies into the ChEA3 server (Keenan et al., 2019). As expected, a highly connected network of transcription factors regulates SALL2 target genes in MGG8TPC (Supplementary Figure 6C). Developmental factors such as SALL3, SOX2, POU3F2, FOXG1, and GLI3 along with the neural transcription factor NPAS3 are tightly connected, suggesting functional cooperativity of these factors with SALL2 targets in MGG8TPC. On the contrary, and consistent with the analysis described above, a poorly connected network is present for the short_E1A target genes, meaning that most of the potential genes regulated by this short isoform in HEK293 cells are not functionally related (Supplementary Figure 6D). The latter poses the question of the role of short_E1A isoform in transcriptional regulation.

Bioinformatic analysis of the short_E1A isoform (198 AA) suggests that it does not contain a classical nuclear localization signal (’pat4,’ ‘pat7’ or ‘bipartite’). Still, according to the PSORT II NNCN score^9^, which discriminates the tendency to be at either the nucleus or the cytoplasm based on the amino acids’ composition, short_E1A has a 52% potential of nuclear localization. Thus, we investigated short_E1A localization by immunocytochemistry and subcellular fractionation experiments. Because HEK293 cells readily express both SALL2 E1 and SALL2 E1A isoforms (Supplementary Figure 7) we used our previously reported HEK293 SALL2 knockout (*SALL2*KO) cell model, which lacks E1 and E1A (Hermosilla et al., 2018) and lentiviral particles carrying a FLAG-short_E1A isoform expression vector. By immunofluorescence analysis, the ectopic short_E1A isoform displayed an average of 85% of cytoplasmic localization (FLAG, Figure 2D). Cell fractionation and Western blot analysis confirmed that the short_E1A isoform is detected mostly in the cytoplasm (FLAG, Figure 2E). Conversely, the FLAG-long E1A isoform expressed in the HEK293 *SALL2*KO cell model displayed near 90% nuclear localization (FLAG, Figure 2F), a trend also seen with endogenous SALL2 isoforms in HEK293 cells (Supplementary Figure 8). These experiments indicate that the short_E1A isoform is mostly cytoplasmic and suggest a small proportion of short_E1A recruits in the chromatin.

### The Long SALL2 E1 and E1A Isoforms Strongly Bind Chromatin, but Binding Motifs Largely Differ by Cell Type

**FIGURE 3:**
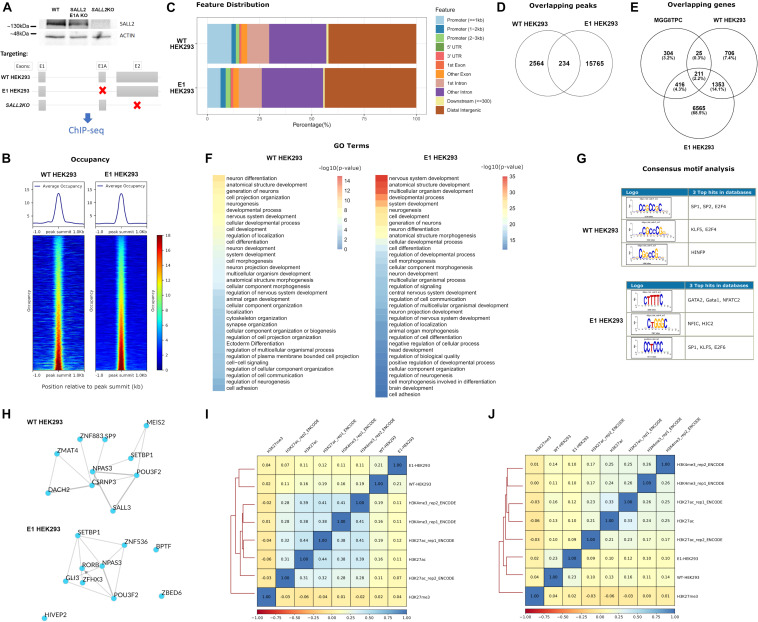
**(A)** (Top) Representative western blot for SALL2 and ACTIN in HEK293 individual clones transfected with Control CRISPR (WT, without sgRNA), SALL2 E1A CRISPR (SALL2 E1A KO), or E2 CRISPR (*SALL2*KO) as indicated. **(Bottom)** Experimental outline of the ChIP-seq experiment. SALL2 Wild-type, SALL2 E1A KO and *SALL2*KO HEK293 cells (denoted as “WT HEK293,” “E1 HEK293” and “*SALL2KO*” ChIP-seq, respectively) were collected and subjected to ChIP-seq analysis as described in material and methods. Gray boxes represent exons, and red crosses indicate targeted exons in each model. **(B)** Average occupancy and heatmap of the occupancy profiling from WT HEK293 and E1 HEK293 experiments. Regions were sorted by occupancy values from the smallest (top) to highest (bottom) and each row represents a 1-kb window focused on the peak centers in the human genome (hg19 build). (Left) WT HEK293 ChIP-seq profiling plotted across 2795 peak regions obtained with MACS2 peak calling software, using *SALL2KO* ChIP-seq as background control. (Right) E1 HEK293 ChIP-seq profiling plotted across 15983 peak regions obtained with MACS2 peak calling software, using *SALL2KO* ChIP-seq as background control. BigWig files used in both plots were generated using a 1x-normalized method against hg19 build, and values were plotted in the heatmaps using the same color scale for comparison purposes. **(C)** Feature distribution of the binding sites of WT HEK293 and E1 HEK293 ChIP-seq datasets (in percentage) upstream and downstream from the TSS of genes. Denoted with distinctive colors are promoters, UTR, and exon regions. **(D)** Venn diagram of the overlapping peaks between WT HEK293 and E1 HEK293 ChIP-seq datasets. **(E)** Venn diagram of the overlapping genes between WT HEK293 and E1 HEK293 ChIP-seq datasets and MGG8TPC dataset. **(F)** Functional enrichment of WT HEK293 and E1 HEK293 ChIP-seq datasets obtained with the gProfiler server (https://biit.cs.ut.ee/gprofiler/gost). To select enriched Gene Ontology (GO) terms, a Bonferroni corrected *p*-value of 0.05 was used as a threshold, and the -log10(*p*-value) was plotted in each dataset. **(G)** Top three over-represented oligos across WT HEK293 and E1 HEK293 ChIP-seq datasets were obtained with the RSAT program (http://rsat.sb-roscoff.fr). The oligos were selected based on *p*-value and recurrence across nucleotide sequences from peaks. **(H)** Transcription factor (TF) local networks constructed with the overrepresented TF from the gene lists in the two ChIP-seq studies (WT HEK293 and E1 HEK293, respectively), obtained with the ChEA3 server (https://amp.pharm.mssm.edu/chea3). **(I)** Spearman pairwise correlation coefficients of binding levels between WT HEK293 ChIP-seq, E1 HEK293 ChIP-seq, and various histone marks in HEK293 cells across the 2795 peak regions deduced from WT HEK293 ChIP-seq experiment. H3K27ac/H3K4me3 corresponds to transcriptionally activating histone marks, and H3K27me3 corresponds to a transcriptionally repressive histone mark. Boxes are coloured according to Spearman correlation coefficients (see legend at the bottom of the graph). **(J)** Same as **(I)** for the 15983 peak regions from the E1 HEK293 ChIP-seq experiment.

The isoform complexity in the SALL2 MGG8TPC ChIP-seq study is not clear (Suvá et al., 2014). Although substantial contribution of E1 and E1A isoforms are expected at the transcriptional level, Figure 1C showed that the SALL2 E1 isoform is downregulated in GBM. Nevertheless, to explore E1 and E1A isoforms’ contribution, we performed ChIP-seq of endogenous SALL2 isoforms in HEK293 cells using a reported anti SALL2 antibody (Bethyl lab), previously used for ChIP experiments (Suvá et al., 2014; Escobar et al., 2015; Ye et al., 2019). We knocked out isoforms driven by the E1A promoter in HEK293 cells using CRISPR/Cas9 as previously reported (Hermosilla et al., 2018; Farkas et al., 2019) and used SALL2 total knockout as background (For validation of SALL2 E1A KO model, see Supplementary Figures 2, 3). In this model, the mRNA expression of total SALL2 and SALL2-E1A diminished compared to the SALL2 wild-type cells without apparent compensation of E1 isoform. As expected, the well-characterized SALL2 target *CDKN1A* (p21) (Li et al., 2004) diminished its levels in the SALL2 E1A-KO compared to the SALL2 wild-type model (Supplementary Figure 3). Thus, we perform ChIP-seq of wild-type HEK293 cells harboring both SALL2 isoforms (hereafter WT-HEK293 ChIP-seq), ChIP-seq of E1-driven promoter isoforms by using SALL2 E1A knockout cells (hereafter E1-HEK293 ChIP-seq), and background ChIP-seq using HEK293 *SALL2*KO model (KO-HEK293 ChIP-seq) (Figure 3A).

The normalized average occupancy around peaks from the WT and E1-HEK293 ChIP-seq datasets indicated that the SALL2 isoforms strongly bind above the background (Figure 3B). Similar to endogenous SALL2 in MGG8TPC, most peaks of these isoforms span introns/intergenic regions (Figure 3C). Around 8% of the WT-HEK293 ChIP-seq peaks overlapped with peaks from the E1-HEK293 ChIP-seq (Figure 3D).

To integrate all ChIP-seq analyses, we constructed a Venn diagram to identify target genes shared between our datasets, and those from the SALL2 ChIP-seq in MGG8TPC (Figure 3E). As expected, between the WT and E1-HEK293 ChIP-seq many target genes were shared (1564 genes, Supplementary Table 4). Around 15% of these overlapped genes were also SALL2 targets in MGG8TPC (211 genes, Figure 3E). These analyzes suggest that long SALL2 isoforms control a core of conserved target genes, regardless of cell type. Similar as the enriched GO terms observed in MGG8TPC ChIP-seq (Supplementary Figure 6A), gene ontology analysis revealed various neural functions of the endogenous long SALL2 isoforms in HEK293 cells (Figure 3F). However, unlike the binding pattern of SALL2 from the MGG8TPC ChIP-seq analysis, the endogenous SALL2 isoforms in HEK293 cells bound GC-rich motifs, corresponding to those of the SP1 family of transcription factors, as previously reported for SALL2 (Gu et al., 2011) (Figure 3G). The binding pattern in HEK293 cells suggests that SALL2 isoforms are not recruited as a complex with SOX/POU family of transcription factors as in MGG8TPC. Noteworthy, similar as in MGG8TPC, wild-type SALL2 isoforms in HEK293 cells exhibited a network of transcription factors consisting of SALL3, POU3F2, and NPAS3 but not SOX2 (see WT in Figure 3H, compared with Supplementary Figure 6C). Interestingly, the E1-HEK293 network is more similar to the MGG8TPC than the WT-HEK293 network (see E1 in Figure 3H, compared Supplementary Figure 6C). We found that like in the MGG8TPC, SOX2 is expressed in mouse brain tissue and P19 embryonic carcinoma cells but not in HEK293 cells (Supplementary Figure 9). Thus, the absence of SOX2 in HEK293 cells might explain the difference in SALL2 binding motifs and networks identified in MGG8TPC and HEK293 cells. Overall, these analyses suggest that SALL2 E1 and/or E1A are the main isoforms binding SALL2 target genes in MGG8TPC ChIP study (Suvá et al., 2014), and SOX2 is a critical factor for maintaining a tight-connected SALL2 transcriptional network in MGG8TPC. We finally asked whether SALL2 long isoforms binding profile correlates with activating and/or repressing histone marks in HEK293 cells using ChIP-seq data of H3K27ac, H3K4me3, and H3K27me3 histone marks submitted by ENCODE and other independent laboratories (see section “Materials and Methods”). Sites bound by SALL2 wild-type (E1 and E1A isoforms) are more closely correlated with histone marks associated with transcriptional activation (Figure 3I, compare H3K27Ac and H3K4me3 versus H3K27me3 spearman correlations). Conversely, sites bound by SALL2 E1 isoform, in the absence of E1A isoform, are more correlated with H3K27me3 than H3K27Ac/H3K4me3 sites (Figure 3J, compare H3K27Ac and H3K4me3 versus H3K27me3 spearman correlations). The latter suggests that in the absence of E1A isoform, E1 isoform binds sites associated with transcriptional repression and reveals a potential suppressive role of E1A in the regulation of SALL2 E1 isoforms in HEK293. These observations also agree with the association of SALL2 E1 canonical isoform with the NuRD repressive complex (Lauberth and Rauchman, 2006).

### *PODXL*, a Potential Novel SALL2 Target Across Tissues and Cancer Types

**FIGURE 4:**
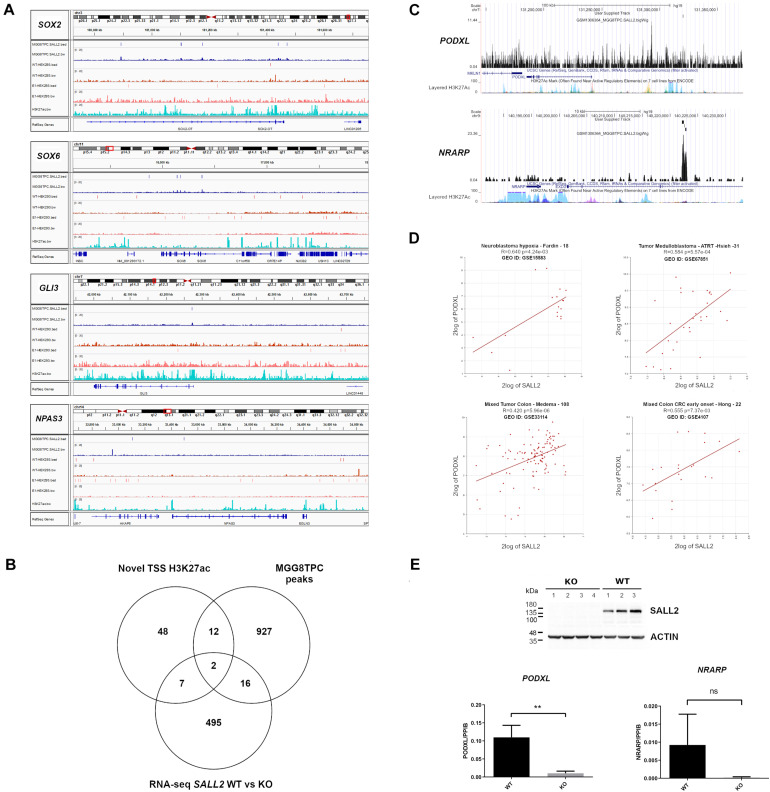
**(A)** BED and Bigwig tracks from *SOX2*, *SOX6*, *GLI3*, and *NPAS3* in the MGG8TPC ChIP-seq dataset showing SALL2 binding enrichment and associated peaks (see tracks at left for description), visualized with IGV. In all graphs, MGG8TPC, WT HEK293, and E1 HEK293 ChIP-seq experiments were included. **(A)** H3K27Ac enhancer mark from ENCODE in HEK293 (GEO: GSE105193) is also shown. **(B)** Venn diagram of MGG8TPC ChIP-seq target genes along with novel H3K27Ac marks around TSS evoked by overexpression of SALL2, POU3F2 and SOX2 in Glioblastoma cells, reported from Suvá et al. (2014) and differentially expressed genes from wild-type, WT (*Sall2 ^+/+^*) versus knockout, KO (*Sall2^– /–^ )* mouse embryonic fibroblasts RNA-seq (GEO: GSE123168). Two core genes were dissected from these comparisons (*PODXL* and *NRARP*). **(C)** Bigwig tracks from *PODXL* and *NRARP* genes in the MGG8TPC ChIP-seq dataset showing SALL2 binding enrichment and associated peaks (see User Supplied Track), plotted with the UCSC genome browser (https://genome.ucsc.edu/, hg19 track). **(D)** Correlation between *SALL2* and *PODXL* expression in brain and colorectal cancers. We used the R2 microarray server (http://r2.amc.nl) to obtain scatter plots of *SALL2* and *PODXL*. We used the algorithm HugoOnce choose a single probe-set to represent every gene and then Pearson’s correlation coefficients (*r*) and associated *P*-values (*p*) were calculated. **(E)** (Top) Detection of SALL2 protein on CCD-841-CoN *SALL2* WT and *SALL2* KO cell clones obtained with CRISPR Cas9 by western blot. Actin is the loading control. (Bottom) mRNA expression of *PODXL* and *NRARP* was evaluated by qPCR analysis using *PPIB* as normalizer. The data correspond to mean ± S.D. from three independent experiments performed in triplicate. Student’s *t*-test determined statistical significance (**P < 0.01, n.s, no significative).

As previously reported for SOX2 and POU3F2 (Suvá et al., 2014), several SALL2 transcriptional network members in MGG8TPC are SALL2 targets. Members from the SOX family of transcription factors such as SOX2 and SOX6, GLI3, and NPAS3 are also SALL2 targets in MGG8TPC (Figure 4A) and in HEK293 cells (Supplementary Table 4), supporting that SALL2 sustains a conserved network of target genes regardless of cell type. To test this hypothesis and prioritize genes involved in tumor development that could be regulated by SALL2, we compared genes nearby H3K27ac sites opened upon overexpression of POU3F2 + SOX2 + SALL2 in differentiated glioblastoma cells (DGC) against SALL2 targets in MGG8TPC cells, both datasets already reported by Suvá et al. (2014) (designated as Novel TSS H3K27ac). We found that fourteen genes are SALL2 targets and are also potentially regulated by SALL2 at the chromatin level (Figure 4B and Supplementary Table 5). Two of these genes, *PODXL* (Podocalyxin-like protein 1) and *NRARP* (NOTCH Regulated Ankyrin Repeat Protein), also found transcriptionally regulated by SALL2 in our previous RNA-seq studies of mouse embryonic fibroblasts (*Sall2 WT* versus *Sall2 KO* models (Farkas et al., 2019), harbor SALL2 ChIP-seq peaks within their regulatory regions in MGG8TPC (Figure 4C). Thus, *PODXL* and *NRARP* may be conserved SALL2 targets across species, tissues, and cancer types. In agreement with the latter, microarray databases showed that *PODXL* and *SALL2* are significantly co-expressed in neuroblastoma and colon cancer samples (see Pearson *p*-values, Figure 4D). Following the microarray data, gene TPM values from MiPanda co-expression analysis demonstrated a high correlation between *SALL2* and *PODXL* in many tissues, including colorectal and brain tissues (see Pearson *p*-values in Supplementary Table 5). To further test this trend, we knocked out SALL2 in the CCD-841-CoN human colon epithelial cell line, where it is endogenously expressed (Figure 4E). *PODXL*, but not *NRARP* mRNA expression, was significantly diminished across several SALL2 knockout clones (*N* = 4) compared with the wild-type counterpart (*N* = 3). Taken together, among the SALL2 target genes functionally related in GBM, *PODXL* might also have a functional relationship with SALL2 in other cancer types. This observation supports a conserved regulatory network for SALL2, surpassing cell type-specific transcriptional regulation.

## Discussion

The SALL2 transcription factor has been associated with various biological processes, including organ development, neuronal differentiation, tumor suppression, cancer progression, and stemness. How SALL2 is involved in such a plethora of actions, some of them even opposites, is still not completely understood. While SALL2 isoforms could explain its different biological functions, most studies about SALL2 targets and functions do not indicate whether E1 or E1A contributes to a specific function. Also, updated information on *SALL2* gene indicates that it has seven protein-coding transcripts composed by a combination of at least five exons (see footnote 1, December 2019) adding more questions of which isoform(s) is (are) predominant in each tissue studied, and which one is (are) responsible for gene expression and function. Here we integrated current data of normal/tumor gene expression databases and spliced RNA-seq data from the ISOexpresso database along with ChIP-seq binding profiles to analyze SALL2 isoforms expression distribution and infer isoform-specific SALL2 targets.

**FIGURE 5:**
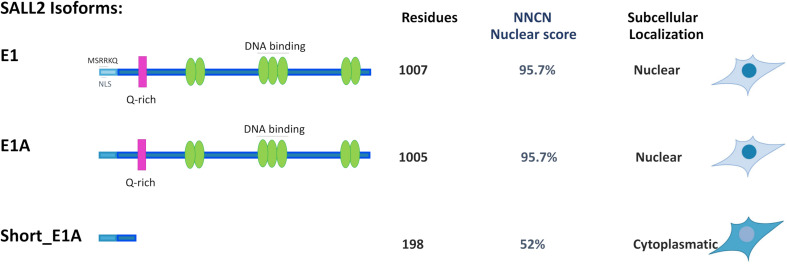
Comparison of Subcellular localization for SALL2 Alternative Isoforms. Representative model of alternative SALL2 isoforms displaying main structural characteristics; green ovals represent C2H2 zinc finger motifs, and the pink rectangle represents the Q-rich sequence present in both E1 and E1A. E1 contains the MSRKKQ motif, found in other SALL members but not in E1A or short E1A isoforms. The number of residues, “NNCN” score, and immunocytochemistry data are indicated.

Our study confirmed that the E1A isoform is the most predominant across tissues and identified a novel short isoform from the E1A-regulated promoter (short_E1A) that is significantly expressed across tissues, even more than the canonical E1 isoform, which is restricted to the brain. Protein subcellular prediction by PSORT II (see footnote 9) indicated that SALL2 E1, E1A and short_E1A isoforms could localize in the nucleus even when canonical nuclear localization signals were not identified. The PSORT II NNCN score, which discriminates the tendency to be at either the nucleus or the cytoplasm based on the amino acids’ composition, found a 52% potential of nuclear localization for the short_E1A, and 95.7% for E1 and E1A isoforms. Different from the short_E1A, long isoforms contain seven C2H2 type zinc finger domain along the sequence and 8.3% content of basic residues (Figure 5). Also, SALL2 E1 contains the negatively charged motif MSRRKQRKPQ, previously associated with transcriptional repression activity of SALL proteins (Lauberth and Rauchman, 2006). Our immunocytochemistry and subcellular fractionation experiments demonstrated that the short_E1A is mainly cytoplasmic, while ectopic SALL2 E1A or endogenous SALL2 protein in HEK293 cells showed a precise nuclear location (Figure 2). Thus, we propose that the ChIP-seq of SALL2 in MGG8TPC and HEK293 cells mainly reflect the E1/E1A nuclear isoforms’ activity while the contribution of short_E1A is marginal.

Interestingly, the short_E1A is significantly expressed across tissues and, of relevance, like the E1A isoform, differentially expressed in some cancer types such as pheochromocytoma, cervical squamous cell carcinoma, or glioblastoma multiforme. Our GO terms analysis of the ChIP-seq experiment performed by the ENCODE consortium suggests that the short_E1A activity in the nucleus is marginal and should be reviewed. However, the differential expression pattern, and functional significance of short_E1A isoform in cancer is intriguing. Some transcription factors splice variants can exert dominant negative (DN) activity hampering the physiological function of long isoforms, associating their expression with different cancer phenotypes. The DN activity of short isoforms can relate to cellular mislocalization and/or sequestration of the functional transcription factor (Belluti et al., 2020). For instance, the Ikaros Family Zinc Finger Protein HELIOS expresses three DN isoforms with cytoplasmic localization in human leukemic T-cell lines, unbalancing the expression of functional variants. The resulting expression of variants alters the transcriptional program, which associates with malignancy. From the same family, IKAROS, a transcription factor that plays a central role in hematopoiesis, presents shorter isoforms that cannot bind DNA and impair the DNA-binding activity of longer IKAROS variants. In this sense, short DN isoforms reduce IKAROS activity in hematopoiesis regulation, correlating with blast crisis development in patients with different types of leukemia (Belluti et al., 2020). Whether the short_E1A isoform falls in any of these categories will require further investigation.

Initial characterization of SALL2 transcriptional activities indicated that it binds to GGG (T/C) GGG consensus sequences (SP1 like) and that the triple zinc finger motif, present in the E1 and E1A isoforms, is required for DNA binding (Gu et al., 2011) (Figure 5). Later, it was demonstrated that SALL2 binds to the expected consensus sequences in promoters of p16 (*CDKN2A)*, *BAX*, *NOXA*, and *c-MYC* genes (Gu et al., 2011; Sung et al., 2012; Escobar et al., 2015; Wu et al., 2015), and GC-box related to the consensus sequences in the p21*CDKN1A* gene promoter (Gu et al., 2011). The activation or repression of these targets by SALL2 in ovarian cancer cells (Li et al., 2004; Gu et al., 2011; Sung et al., 2012), and mouse embryonic fibroblasts (Escobar et al., 2015; Hermosilla et al., 2017, 2018), associate SALL2 with a tumor suppressor function. However, in human embryonic stem cells (hESCs), SALL2 is significantly expressed (Sperger et al., 2003; van den Berg et al., 2010) and interacts with factors that maintain stemness, such as SALL4 (Yang et al., 2019) and SOX2 (Suvá et al., 2014). According to a neural developmental function, SALL2 promotes neurotrophin-mediated neuronal differentiation through NGF/p75NTR/TrkA signaling in rat and mouse cells (Pincheira et al., 2009). Its expression is associated with two subtypes of radial glia cells during brain development (La Manno et al., 2016). In line with its neural and stem cell renewal function, SALL2, combined with SOX2, POU3F2, and OLIG2 transcription factors, promotes the de-differentiation of glioblastoma cells into stem-like tumor propagating cells. In this latter context, opposite to its tumor suppressor role, SALL2 binds to ATTCAT (Sox8 dimeric like) sequences (Suvá et al., 2014). Like the glioblastoma context, SALL2 recently was associated with the de-differentiation of breast and melanoma cancer cells into cancer stem cells together with YB-1 and other transcription factors (Yang et al., 2019). Our binding motif analyses confirmed that in the MGG8TPC context, SALL2 isoforms are primarily recruited to the previously reported A/T rich motifs (SOX and POU like). Interestingly, in the context of the HEK293 cells, endogenous SALL2 is recruited to GC-rich motifs (SP1 like) when E1 and E1A isoforms are present (HEK293 wild-type), but to CT-rich motifs when only SALL2-E1 is present (HEK293 E1A-KO). Together, these results suggest that SALL2 binding to DNA depends on the presence of specific partners or isoforms. In this regard, there is evidence of the interaction between SALL2 and SP1 in human ovarian surface epithelial (HOSE) cells. SALL2 and SP1 associate *in vivo* by binding to DNA with multiple copies of their common or overlapping GC rich motif (Gu et al., 2011). In glioblastoma, instead, SALL2 interacts with SOX2 and binds to A/T rich motifs (Suvá et al., 2014). Since HEK293 do not express SOX2, the presence/absence of this transcription factor could explain the difference in SALL2 binding motifs between MGG8TPC and HEK293 cell contexts. Whether SALL2 binding to DNA depends on the presence of more than one isoform requires further investigation. Regarding the latter, we provided evidence that E1 isoform, in absence of E1A isoform binds sites in the genome associated with transcriptional repression rather than activation, as seen in the wild-type context. Our finding is consisting with previous reports where loss of one splice isoform affects the function of the other splice isoform (Bhuiyan et al., 2018).

Independently of the cell context, we identified a conserved network of transcription factors accompanying SALL2 in regulating transcriptional targets in MGG8TPC and HEK293 cells. From this network, we inferred a conserved transcriptional regulation through SALL3, POU3F2, and NPAS3, with or without SOX2; therefore, there are conserved transcriptional targets. In these comparisons, it was not surprising to see embryonic and neural markers in both datasets due to the proposed neuronal phenotype (neural origin) of HEK293 cells. Previous studies argue that HEK293 and other Ad-transformed HEK lines belong to the neuronal lineage, with an expression pattern like that of a typical early differentiating neuron or neuronal stem cell (Shaw et al., 2002). Cross-validated SALL2 target genes from GBM, our ChIP-seq studies in HEK293 cells, and the available gene expression profiling for SALL2 identified a core set of genes, including *PODXL* and *NRARP*. Consistently, a positive relationship between *SALL2* and *PODXL* expression is found in several tissues, including brain and colon cancers, and *PODXL* expression is perturbed upon CRISPR/Cas9-mediated SALL2 knock-out in a human epithelial colon cell line. Podocalyxin (*PODXL*) is a transmembrane protein belonging to the CD34 family of sialomucins expressed in multiple normal cell types, including podocytes, vascular endothelium, platelets, hematopoietic progenitors, embryonic stem cells, and a subset of neurons (Tamayo-Orbegozo et al., 2020). It is overexpressed in various tumor cell types and is associated with their aggressiveness and poor prognosis (Xu et al., 2018). PODXL upregulation is a cancer stem cell marker in meningiomas (Schulten and Hussein, 2019) and a marker of poor outcome in patients with GBM (Binder et al., 2013). It is proposed as a biomarker of poor prognosis in colorectal cancers also (Larsson et al., 2012, 2013, 2016; Lee et al., 2017). Thus, the SALL2-PODXL transcriptional regulation may be necessary for cancer stemness. Altogether, our analyses suggest that SALL2 has a conserved network of target genes that rely on self-renewal transcription factors and a core of genes important for SALL2-mediated transcriptional regulation in human cancers.

## Data Availability Statement

The generated ChIP-seq datasets for this study can be found in the NCBI GEO repository GSE145940 https://www.ncbi.nlm.nih.gov/geo/query/acc.cgi?acc=GSE145940.

## Author Contributions

CF, RP, and MH designed the study. CF, MH, AQ, VH, CA, and CFA performed the experiments and acquisition of data. CF, MH, AC, and RP performed the overall data interpretation. MH, CFA, and AL performed the ChIP-seq of WT and E1 SALL2 isoforms. CF, AC, and RP wrote the manuscript. MH, AQ, VH, CA, CFA, and AL critically reviewed the manuscript. All the authors read and approved the final version of the manuscript.

## Conflict of Interest

The authors declare that the research was conducted in the absence of any commercial or financial relationships that could be construed as a potential conflict of interest.

## Footnotes

1 https://www.ensembl.org/

2 http://mipanda.org

3 https://cran.r-project.org/web/packages/pheatmap/index.html

4 https://tidyverse.tidyverse.org/

5 https://bioconductor.org/packages/release/bioc/html/ChIPseeker.html

6 http://rsat.sb-roscoff.fr

7 https://www.sanger.ac.uk/science/groups/vertebrate-annotation

8 https://github.com/cfarkas/SALL2_conserved_network_analysis

9 http://psort.hgc.jp/form2.html

## Supplementary Material

Supplementary Figure 1 Workflow of our method to infer SALL2 isoforms distribution, targets, and transcription factor network analysis.

Supplementary Figure 2 (Upper) Sanger sequencing data of SALL2 E1A KO clone (clone 17) compared to a wild type HEK293 clone. Gray highlight indicates the beginning of long SALL2 E1A isoform from the start codon of E1A exon, and bold red letters indicate the protospacer adjacent motif (PAM) sequence targeted by the guide RNA of CRISPR/Cas9. (Lower) Clustal alignment of the referred sequences, depicting the loss of the start codon of SALL2 E1A isoform and multiple subsequent mutations across the Clone 17 sequence.

Supplementary Figure 3 **(A)** Equal amounts of protein lysates from SALL2 WT and SALL2 E1A KO human embryonic kidney HEK293 cells were evaluated for SALL2 and β-actin levels by western blot analysis. **(B)** Quantitative real-time qPCR of SALL2 total mRNA, normalized against *PPIB* (cyclophilin) levels in HEK293 cells. Bars denote standard deviation for three replicates. **(C)** Same as **(B)** for SALL2 E1A isoform. **(D)** Same as **(B)** for SALL2 E1 isoform. **(E)** Same as **(B)** for p21 (*CDKN1A*) gene.

Supplementary Figure 4 *SALL2* mRNA total expression across normal tissues. SALL2 normalized gene counts as transcripts per million (TPM) were plotted across tissues, each denoted with a color. Black discontinued lines correspond to Median TPM values in each tissue.

Supplementary Figure 5 *SALL2* isoform expression across tissues. Normalized gene counts as transcripts per million (TPM) across tissues was plotted from every SALL2 isoform characterized in Ensembl. We used Violin plots to visualize distribution. 75% median, and 25% quartiles are denoted from top to bottom as dashed lines.

Supplementary Figure 6 Integrated analysis of MGG8TPC (GSE54047) and ENCODE short_E1A SALL2 ChIP-seq datasets (GSE105193). **(A)** Gene Ontology functional enrichment of target genes derived from MGG8TPC and short E1A ChIP-seq (ENCODE) publicly available ChIP-seq, obtained with the gProfiler server (https://biit.cs.ut.ee/gprofiler/gost). To select enriched Gene Ontology (GO) terms, a Bonferroni corrected *p*-value of 0.05 was used as a threshold, and the –log10(*p*-value) was plotted in each dataset. **(B)** Top over-represented oligos across ChIP-seq datasets obtained with the RSAT program (http://rsat.sb-roscoff.fr). The oligos were selected based on *p*-value and recurrence across nucleotide sequences from peaks. **(C,D)** Transcription factor (TF) local networks constructed with the overrepresented TF from the gene lists in the two ChIP-seq studies (MGG8TPC and short_E1A, respectively), obtained with the ChEA3 server (https://amp.pharm.mssm.edu/chea3).

Supplementary Figure 7 Tissue-dependent SALL2 isoforms expression. Isoform-specific primers used for RT-PCR are the same from Ma et al. (2001). Products of the two primer pairs were specific for *E1A* (383 bp) and E1 (356 bp). Comparable amounts of cDNAs were utilized. PCR products were electrophoretically separated on 2% polyacrylamide gels. β-ACTIN was used as loading control. Expression was evaluated in MCF7 breast cancer cells, HCT116 and HM7 colon cancer cells, H1299 lung cancer cells and HEK293 embryonic kidney cells.

Supplementary Figure 8 **(A)** Immunofluorescence of endogenous SALL2 (red) detected with SALL2 antibody (SIGMA) in wild type HEK293 cells. White arrows depict nuclear SALL2, and the arrowhead shows cytoplasmic SALL2. White dashed lines show cell limits. **(B)** Hoechst Nuclear staining (green) of **(A)**. **(C)** Merge between SALL2 (red) and Hoechst (green) signals of **(A)**. **(D)** Bright field of **(C)**, showing cell cytoplasm and boundaries. White bars denote 20 μM.

Supplementary Figure 9 **(A)** Equal amount of protein lysates from P19 mouse embryonic carcinoma and human embryonic kidney HEK293 cells were evaluated for SALL2, SOX2, and HDAC1 levels by western blot analysis. **(B)** Protein lysates from HEK293 and mouse embryos brain tissues (E13.5) *Sall2^+/+^* and *Sall2*^–/–^ were evaluate for SALL2 and SOX2 levels as in **(A)**. ACTIN is the loading control. Representative western blots are shown. The analysis shows that SOX2 is not present in HEK293 cells.

Supplementary Table 1 List of oligos used for human E1A Cas9 targeting, *SALL2* related DNA sequencing, and qPCR of specific genes.

Supplementary Table 2 The raw TPM values for *SALL2* mRNA in normal and cancer tissues, including statistical comparisons (*p*-values) from Student *t*-test.

Supplementary Table 3 Short_E1A (GSE105193) and MGG8TPC (GSE54047) ChIP-seq annotations with R package ChIPseeker, including overlap and GO terms for each experiment.

Supplementary Table 4 WT and E1 HEK293 ChIP-seq (GSE145940) annotation with R package ChIPseeker, including the overlap between these datasets against MGG8TPC experiment. GO terms for each experiment were obtained by using gProfiler.

Supplementary Table 5 Overlap of Novel TSS evoked by SALL2 overexpression in MGG8TPC cells, captured by H3K27ac ChIP-seq against differentially expressed genes upon *Sall2* loss in Mouse embryonic fibroblasts (GSE123168) and SALL2 peaks in MGG8TPC. The *SALL2* and *PODXL* correlation across tissues were obtained with MiPanda server.
