# Supplementary figures and images for: Characterization of SALL2 Gene Isoforms and Targets Across Cell Types Reveals Highly Conserved Networks
Source: Front Genet. 2021 Feb 22;12:613808. doi: 10.3389/fgene.2021.613808 (PMC7937961; doi:10.3389/fgene.2021.613808)

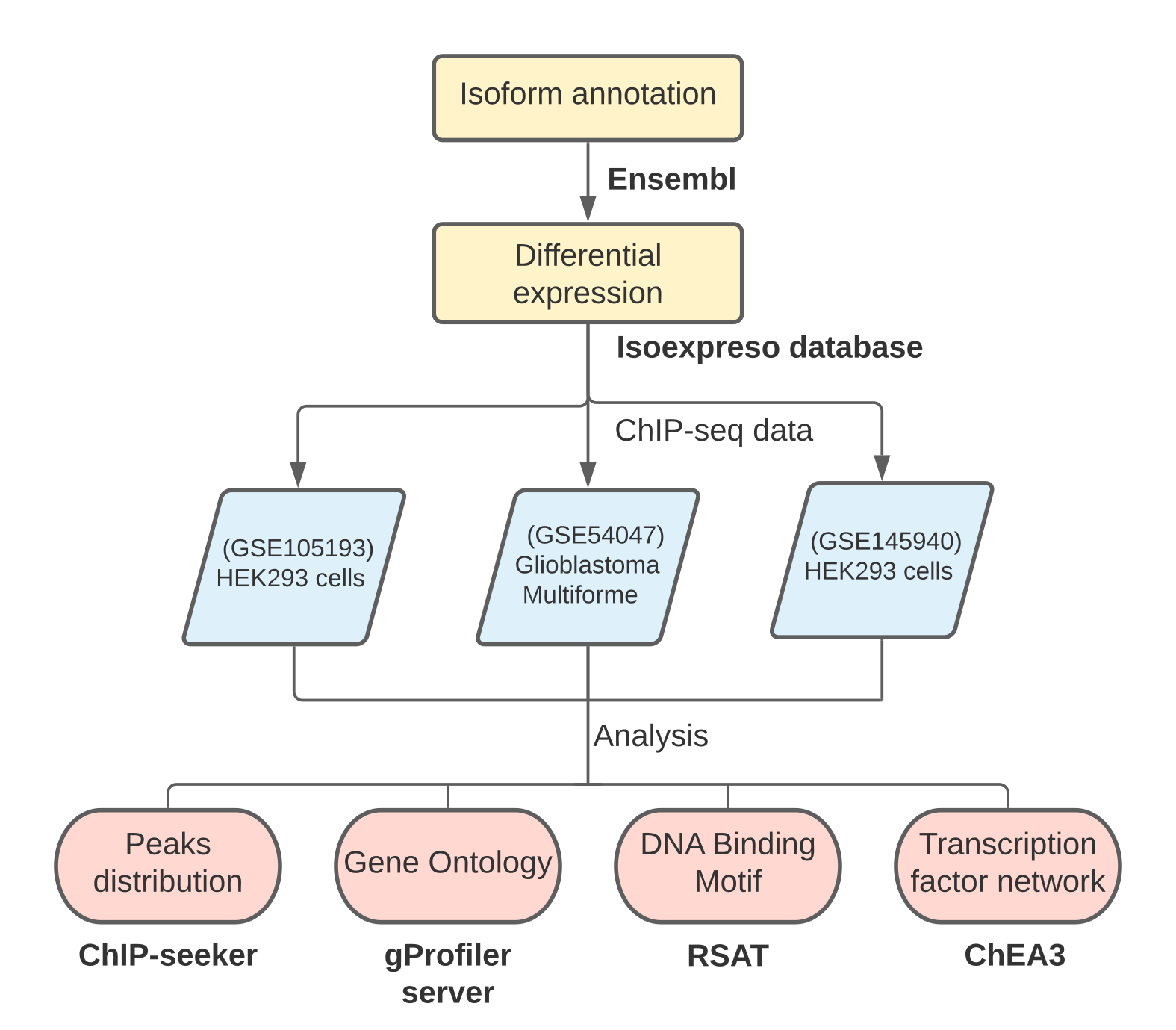

Supplement: Supplementary Figure 1 — Workflow of our method to infer SALL2 isoforms distribution, targets, and transcription factor network analysis. [file Image_1.tif]

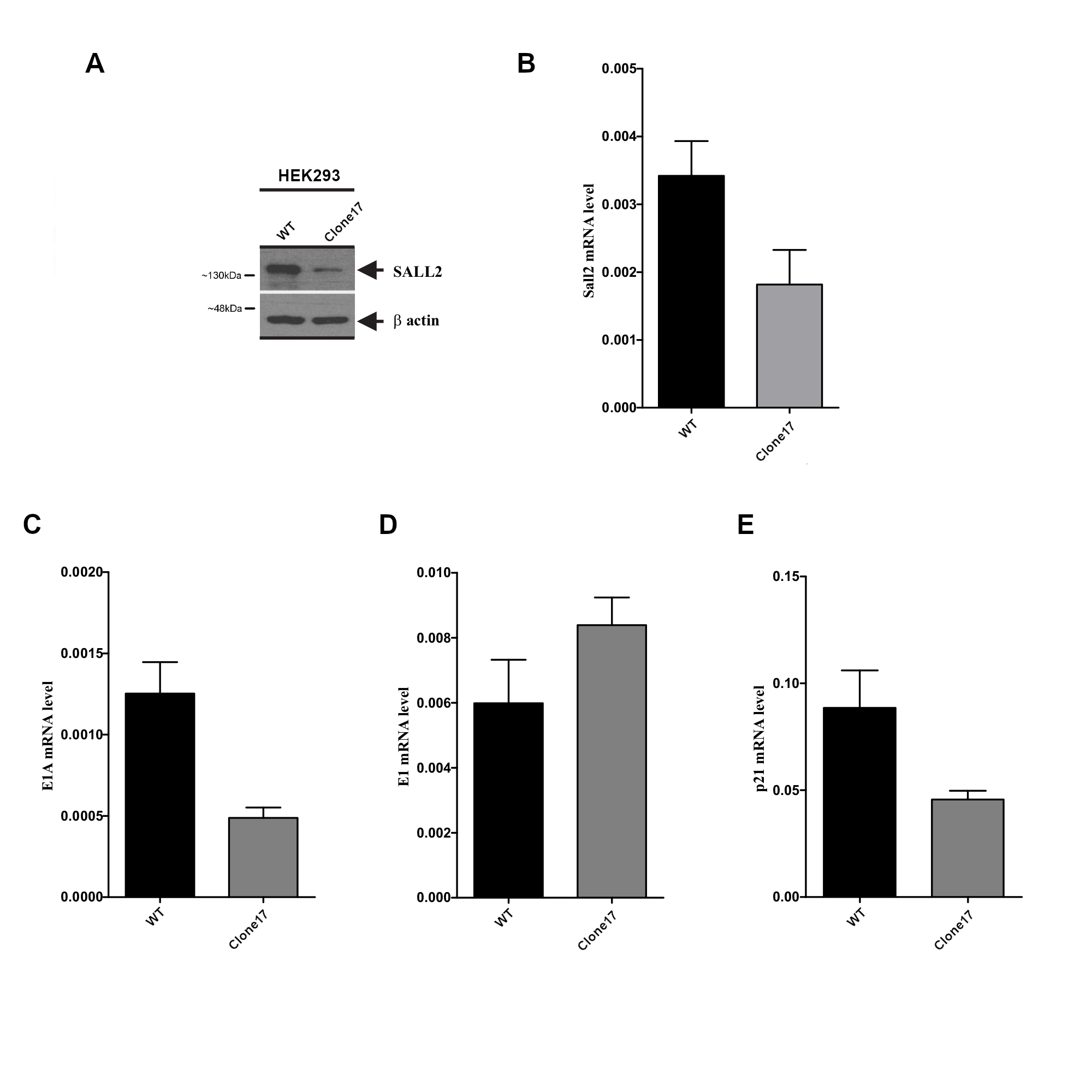

Supplement: Supplementary Figure 3 — (A) Equal amounts of protein lysates from SALL2 WT and SALL2 E1A KO human embryonic kidney HEK293 cells were evaluated for SALL2 and β-actin levels by western blot analysis. (B) Quantitative real-time qPCR of SALL2 total mRNA, normalized against PPIB (cyclophilin) levels in HEK293 cells. Bars denote standard deviation for three replicates. (C) Same as (B) for SALL2 E1A isoform. (D) Same as (B) for SALL2 E1 isoform. (E) Same as (B) for p21 (CDKN1A) gene. [file Image_3.tif]

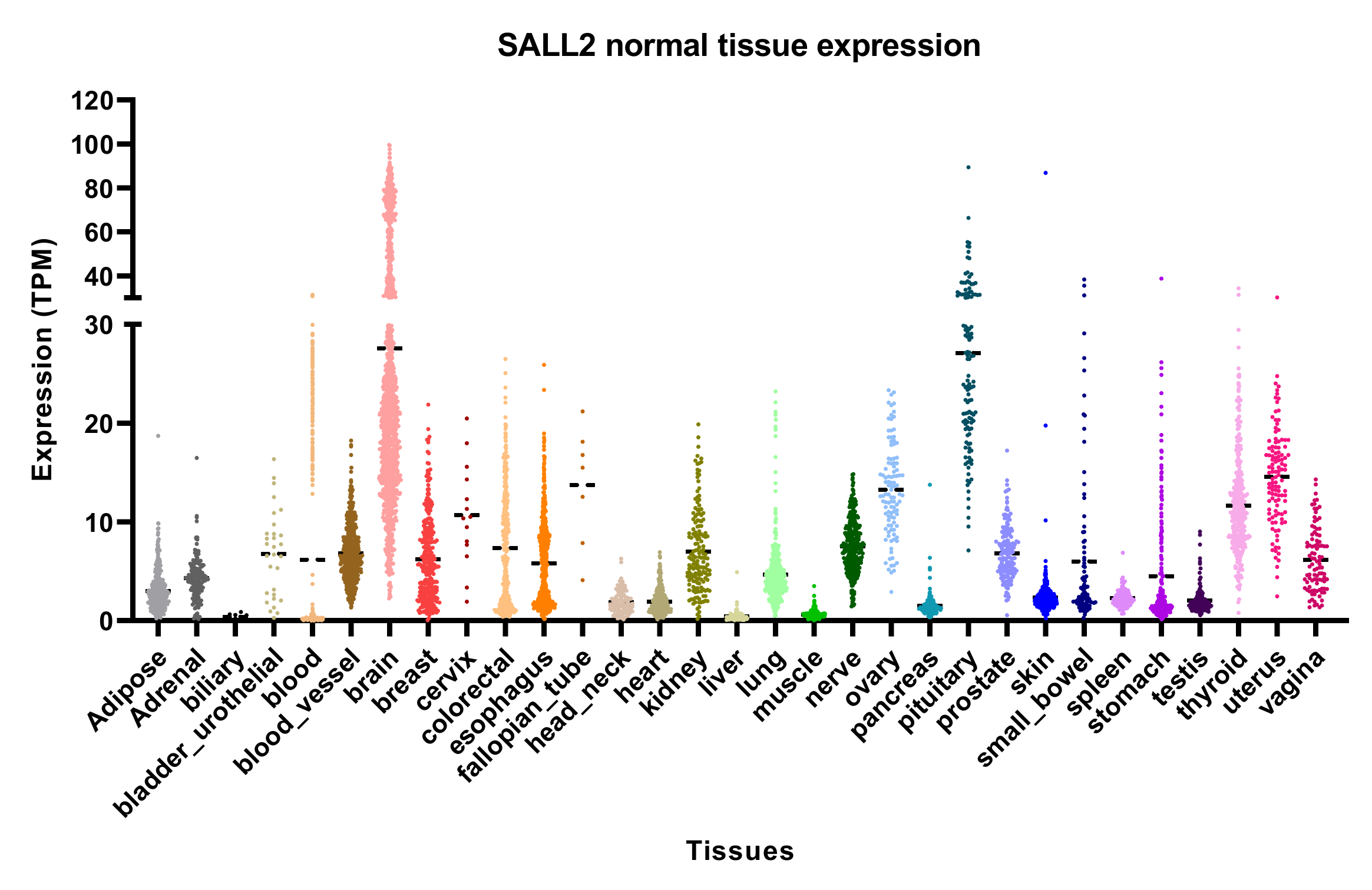

Supplement: Supplementary Figure 4 — SALL2 mRNA total expression across normal tissues. SALL2 normalized gene counts as transcripts per million (TPM) were plotted across tissues, each denoted with a color. Black discontinued lines correspond to Median TPM values in each tissue. [file Image_4.tif]

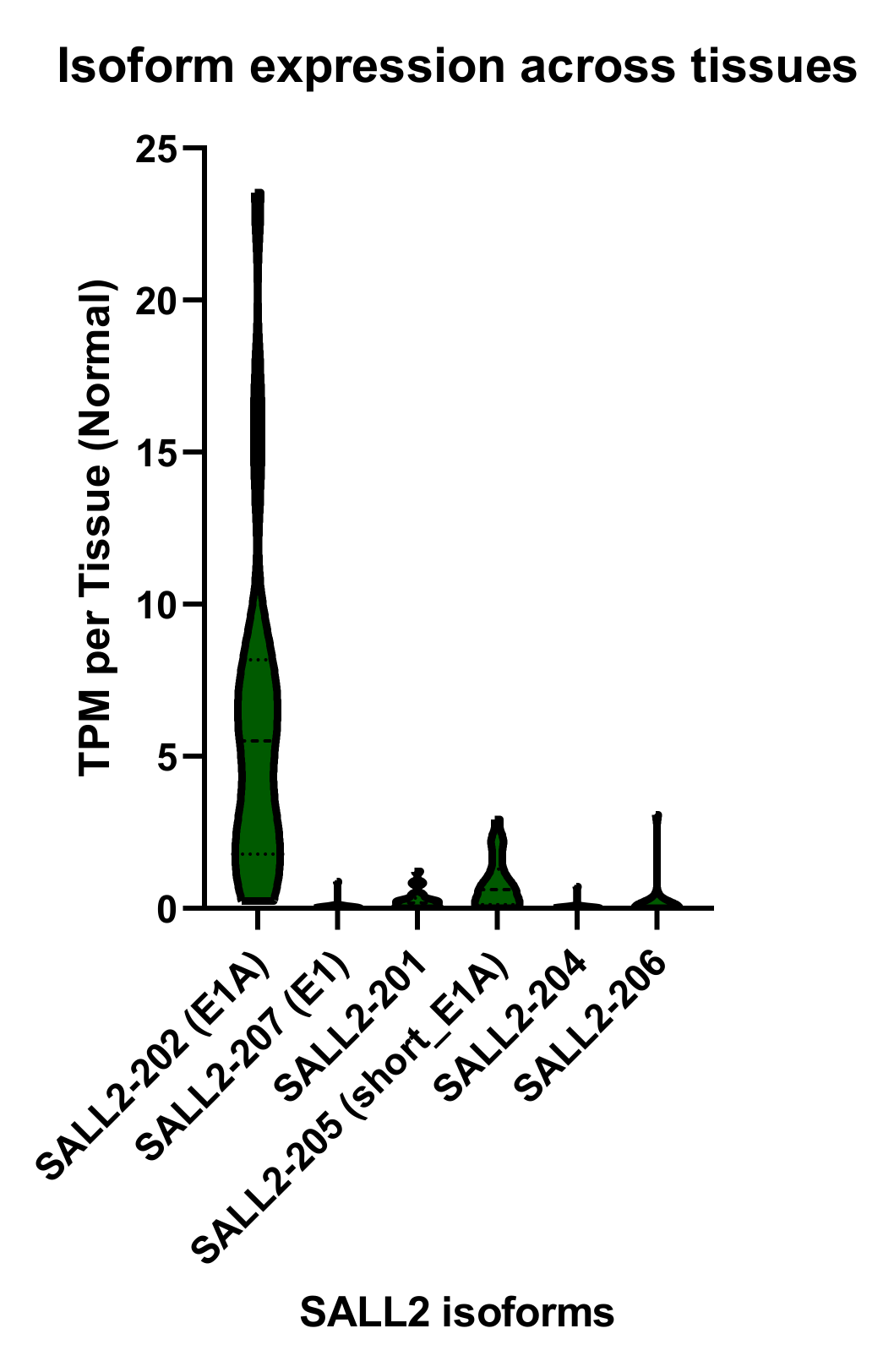

Supplement: Supplementary Figure 5 — SALL2 isoform expression across tissues. Normalized gene counts as transcripts per million (TPM) across tissues was plotted from every SALL2 isoform characterized in Ensembl. We used Violin plots to visualize distribution. 75% median, and 25% quartiles are denoted from top to bottom as dashed lines. [file Image_5.tif]

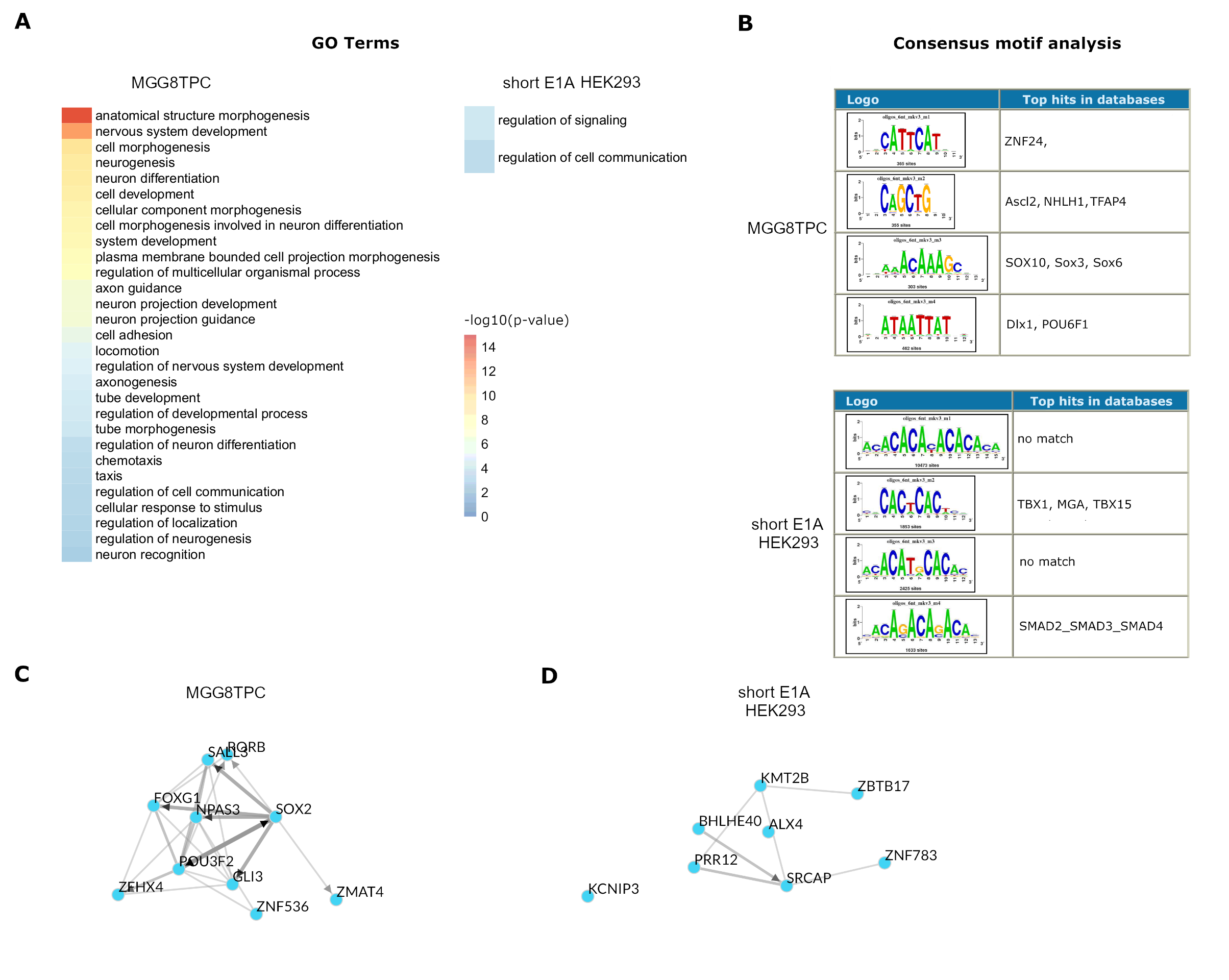

Supplement: Supplementary Figure 6 — Integrated analysis of MGG8TPC (GSE54047) and ENCODE short_E1A SALL2 ChIP-seq datasets (GSE105193). (A) Gene Ontology functional enrichment of target genes derived from MGG8TPC and short E1A ChIP-seq (ENCODE) publicly available ChIP-seq, obtained with the gProfiler server (https://biit.cs.ut.ee/gprofiler/gost). To select enriched Gene Ontology (GO) terms, a Bonferroni corrected p-value of 0.05 was used as a threshold, and the –log10(p-value) was plotted in each dataset. (B) Top over-represented oligos across ChIP-seq datasets obtained with the RSAT program (http://rsat.sb-roscoff.fr). The oligos were selected based on p-value and recurrence across nucleotide sequences from peaks. (C,D) Transcription factor (TF) local networks constructed with the overrepresented TF from the gene lists in the two ChIP-seq studies (MGG8TPC and short_E1A, respectively), obtained with the ChEA3 server (https://amp.pharm.mssm.edu/chea3). [file Image_6.tif]

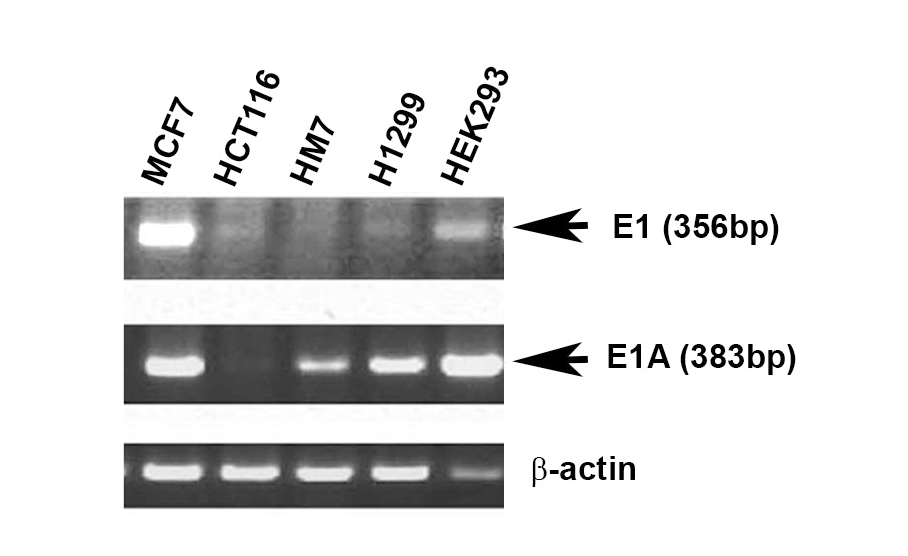

Supplement: Supplementary Figure 7 — Tissue-dependent SALL2 isoforms expression. Isoform-specific primers used for RT-PCR are the same from Ma et al. (2001). Products of the two primer pairs were specific for E1A (383 bp) and E1 (356 bp). Comparable amounts of cDNAs were utilized. PCR products were electrophoretically separated on 2% polyacrylamide gels. β-ACTIN was used as loading control. Expression was evaluated in MCF7 breast cancer cells, HCT116 and HM7 colon cancer cells, H1299 lung cancer cells and HEK293 embryonic kidney cells. [file Image_7.tif]

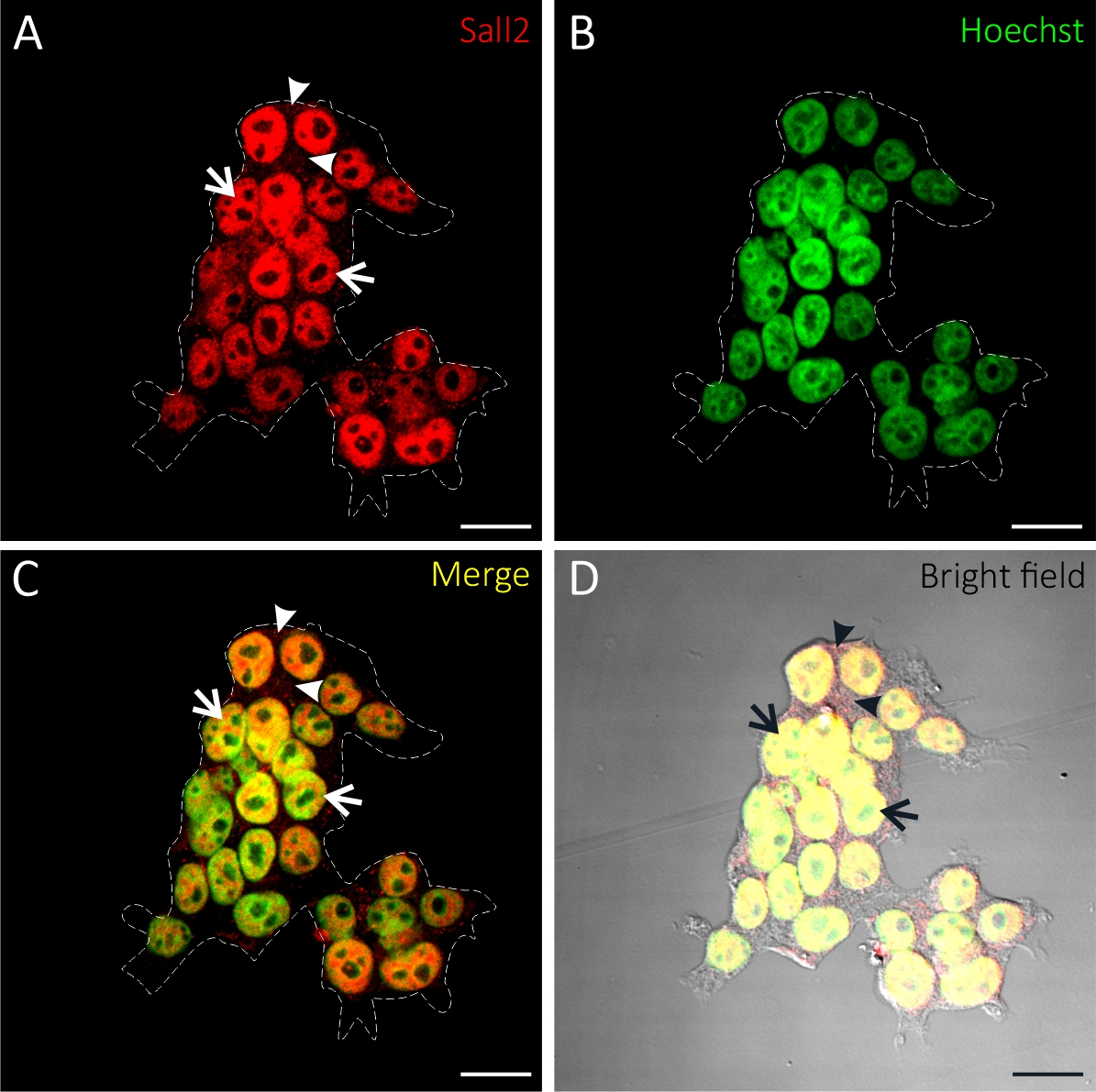

Supplement: Supplementary Figure 8 — (A) Immunofluorescence of endogenous SALL2 (red) detected with SALL2 antibody (SIGMA) in wild type HEK293 cells. White arrows depict nuclear SALL2, and the arrowhead shows cytoplasmic SALL2. White dashed lines show cell limits. (B) Hoechst Nuclear staining (green) of (A). (C) Merge between SALL2 (red) and Hoechst (green) signals of (A). (D) Bright field of (C), showing cell cytoplasm and boundaries. White bars denote 20 μM. [file Image_8.tif]

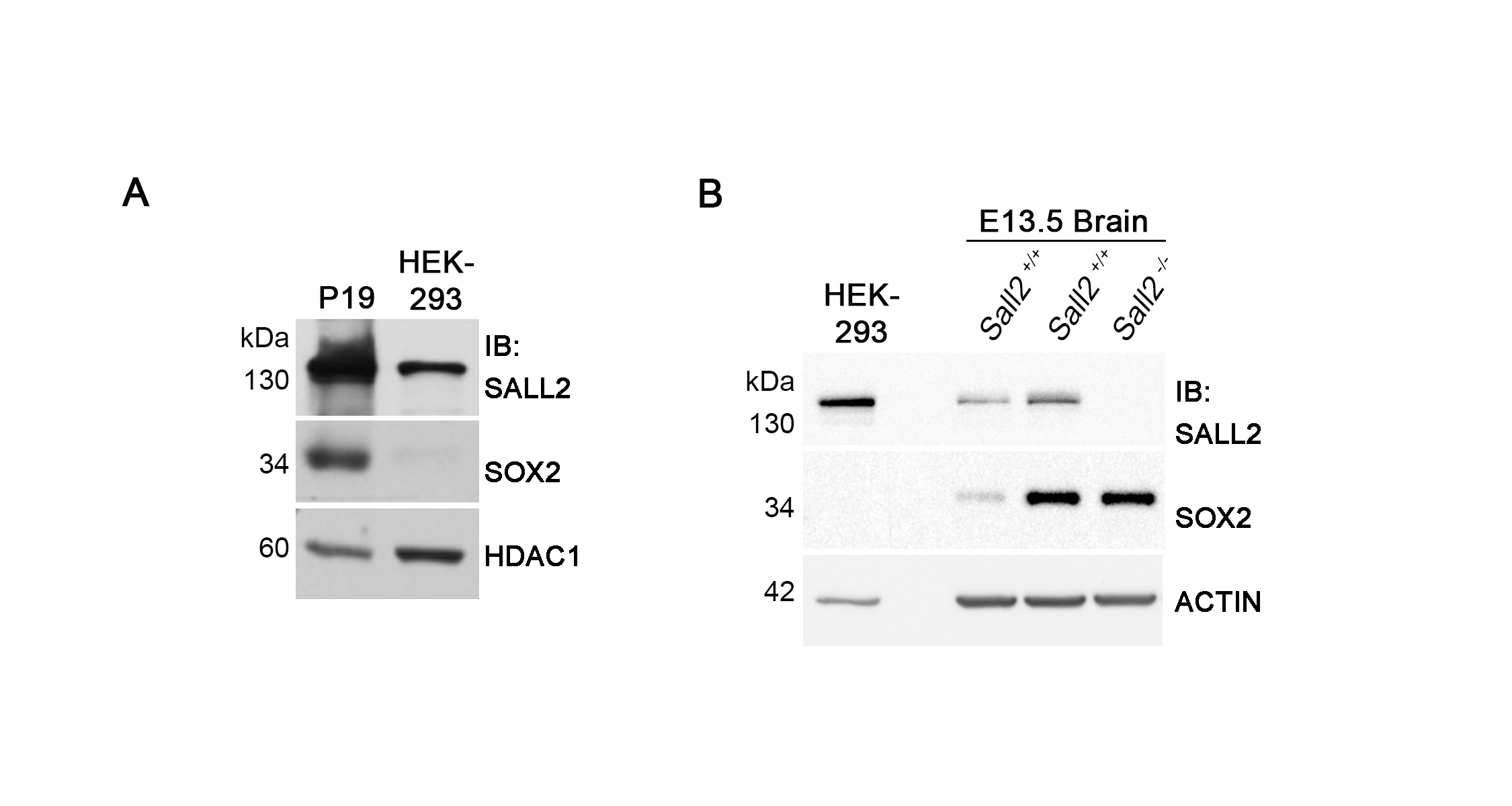

Supplement: Supplementary Figure 9 — (A) Equal amount of protein lysates from P19 mouse embryonic carcinoma and human embryonic kidney HEK293 cells were evaluated for SALL2, SOX2, and HDAC1 levels by western blot analysis. (B) Protein lysates from HEK293 and mouse embryos brain tissues (E13.5) Sall2+/+ and Sall2–/– were evaluate for SALL2 and SOX2 levels as in (A). ACTIN is the loading control. Representative western blots are shown. The analysis shows that SOX2 is not present in HEK293 cells. [file Image_9.tif]
