# Supplementary material for: Characterization of SALL2 Gene Isoforms and Targets Across Cell Types Reveals Highly Conserved Networks
Source: Front Genet. 2021 Feb 22;12:613808. doi: 10.3389/fgene.2021.613808 (PMC7937961; doi:10.3389/fgene.2021.613808)
Supplement: Supplementary Figure 2 — (Upper) Sanger sequencing data of SALL2 E1A KO clone (clone 17) compared to a wild type HEK293 clone. Gray highlight indicates the beginning of long SALL2 E1A isoform from the start codon of E1A exon, and bold red letters indicate the protospacer adjacent motif (PAM) sequence targeted by the guide RNA of CRISPR/Cas9. (Lower) Clustal alignment of the referred sequences, depicting the loss of the start codon of SALL2 E1A isoform and multiple subsequent mutations across the Clone 17 sequence. [file Image_2.pdf]

>WT
NNNTATNNTANNTAGAGGCTGCCGCAGACCCAGCGGCCATCGCCTCCCTCAATCCCAATATCCATTGTCTCCCACCCCACC
TGGCCCTCCACTT**CCCCCACAA**CCATGGCGCACGAATCCGAGAGGAGCTCTCGTCTCGGGGTGCCCTGCGGGGAGCCGGCA
GAGCTCGGAGGTGCGGTAGGGTCGGTCGGGCTGGGCAGGGGTGGGGGCGGAGGGGGATGCGCAGGGGCGGCGAAGACTTTC
GGTGGGTGGGAGAAAATGCANGNNNNNNNNGTNNNNNNNNNN

= MAHESERSSRLGVPCGEPAELGGAVGSVGLGRGGGGGGCAGAAKTFGGWEKM

>Clone_17
TAGNTAGAGGCTGCCGCATAGCCAGCGGCCATCGCCTCCCTCAATTCCAATATCCATTTTCTCCCACCCCTCGTGTCCCTC
CCCTT**CCCCC**CCAAGGCGCNCGAATCCGAGAGGAGCTCTCGTCTCGGGGTGCCCTGCGGGGAGCCGGCAGAGCTCGGAGGT
GCGGTAGGGTCGGTCGGGCTGGGCAGGGGTGGGGGCGGAGGGGGAATGCGCAAGGGGCGGGCGAAGACTTTCGGTGGGTGG
NNNNNNNANNGCNNNNNNNNCNNNNNNNNNNNNNNNNN

= KAXESERSSRLGVPCGEPAELGGAVGSVGLGRGGGGGGMRKGRAKTFGGW

```
WT              NNNTATNNTANNTAGAGGCTGCCGCAGACCCAGCGGCCATCGCCTCCCTCAATCCCAATA  60
Clone_17        --------TAGNTAGAGGCTGCCGCATAGCCAGCGGCCATCGCCTCCCTCAATTCCAATA  52
                        ** *************** * ************************ ******

WT              TCCATTGTCTCCCACCCCACCTGGCCCTCCACTTCCCCCACAACCATGGCGCACGAATCC  120
Clone_17        TCCATTTTCTCCCACCCCTCGTGTCCCTCCCCTTCCCCC----CCAAGGCGCNCGAATCC  108
                ****** *********** * ** ****** ********    *** ***** *******

WT              GAGAGGAGCTCTCGTCTCGGGGTGCCCTGCGGGGAGCCGGCAGAGCTCGGAGGTGCGGTA  180
Clone_17        GAGAGGAGCTCTCGTCTCGGGGTGCCCTGCGGGGAGCCGGCAGAGCTCGGAGGTGCGGTA  168
                ************************************************************

WT              GGGTCGGTCGGGCTGGGCAGGGGTGGGGGCGGAGGGGGATGCGCAGGG---GCGGCGAAG  237
Clone_17        GGGTCGGTCGGGCTGGGCAGGGGTGGGGGCGGAGGGGGAATGCGCAAGGGGCGGGCGAAG  228
                ***************************************        *     *******

WT              ACTTTCGGTGGGTGGGAGAAAATGCANGNNNNNNNNGTNNNNNNNNNN----- 285
Clone_17        ACTTTCGGTGGGTGGNNNNNNNANNGCNNNNNNNNCNNNNNNNNNNNNNNNNN 281
                ***************             *******   **********
```
